# How can we improve specialist health services for children with multi-referrals? Parent reported experience

**DOI:** 10.1186/s12913-020-05666-9

**Published:** 2020-08-24

**Authors:** Ragnhild B. Lygre, Viktoria Mellingen Thuen, Rolf Gjestad, Tone M. Norekvål, Gottfried Greve, Thomas Mildestvedt, Irene Bircow Elgen

**Affiliations:** 1 Department of Child and Adolescent Mental Health Services, Haukeland University Hospital, Bergen, Norway; 2 Department of Clinical Medicine, University of Bergen, Bergen, Norway; 3 Research Department, Division of Psychiatry, Haukeland University Hospital, Bergen, Norway; 4 Centre for Research and Education in Forensic Psychiatry, Haukeland University Hospital, Bergen, Norway; 5 Centre on Patient-reported Outcomes Data, Haukeland University Hospital, Bergen, Norway; 6 Department of Clinical Science, University of Bergen, Bergen, Norway; 7 Department of Heart Disease, Haukeland University Hospital, Bergen, Norway; 8 Department of Global Public Health and Primary Care, University of Bergen, Bergen, Norway

**Keywords:** Multimorbidity, Non-specific condition, Multi-referral, Pediatrics, Mental health care, Health service research, Patient reported experience measures, Complementary and integrative health

## Abstract

**Background:**

Children with combined mental and somatic conditions pose a challenge to specialized health services. These cases are often characterized by multi-referrals, frequent use of health services, poor clinical and cost effectiveness, and a lack of coordination and consistency in the care. Reorganizing the health services offered to these children seems warranted. Patient reported experiences give important evidence for evaluating and developing health services. The aim of the present descriptive study was to explore how to improve specialist health services for children with multiple referrals for somatic and mental health conditions. Based on parent reported experiences of health services, we attempted to identify key areas of improvement.

**Methods:**

As part of a larger, ongoing project; “Transitioning patients’ Trajectories”, we asked parents of children with multiple referrals to both somatic and mental health departments to provide their experiences with the services their children received. Parents/guardians of 250 children aged 6–12 years with multi-referrals to the Departments of Pediatrics and Child and Adolescent Mental Health at Haukeland University Hospital between 2013 and 2015 were invited. Their experience was collected through a 14 items questionnaire based on a generic questionnaire supplied with questions from parents and health personnel. Possible associations between overall experience and possible predictors were analyzed using bivariate regression.

**Results:**

Of the 250 parents invited, 148 (59%) responded. Mean scores on single items ranged from 3.18 to 4.42 on a 1–5 scale, where five is the best possible experience. In the multiple regression model, perception of wait time (*r* = .56, *CI =* .44–.69 / β = 0.16, CI = .05–.28), accommodation of consultations (*r =* .71, *CI* = .62–.80 / β = 0.25, CI = .06–.45 / β = 0.27, CI = .09–.44), providing adequate information about the following treatment (*r* = .66, *CI* = .55–.77 / β = 0.26, CI = .09–.43), and collaboration between different departments at the hospital (*r* = .68, CI = .57–.78 / β = 0.20, CI = -.01–.40) were all statistically significantly associated with parents overall experience of care.

**Conclusions:**

The study support tailored interdisciplinary innovations targeting wait time, accommodation of consultations, communication regarding the following treatment and collaboration within specialist health services for children with multi-referrals to somatic and mental specialist health care services.

## Background

Children with multiple hospital referrals are often lacking tailored and coordinated specialist support [1]. The lack of clarification of conditions and available treatment options, is a potential burden for both patients and their families, as well as health care personnel and society.

Today’s health services predominantly treat body and mind as two separate entities, separating physical from mental health. Emerging evidence from both medical and psychological research, however, suggests that mind and body are inextricably bound [2, 3]. Several studies indicate high rates of co-occurrence of physical and mental health complaints in childhood [2] with similar etiology [3], and often resulting in non-specific conditions. Combined mental and physical conditions can emerge as comorbidity, multimorbidity or several coexisting diffuse, but debilitating, health complaints. Subthreshold mental and physical health complaints that do not meet the diagnostic criteria for a specific diagnosis, could still have a high degree of complexity and cause severely impaired function in the child. Such non-specific conditions contain symptoms or manifestations that are difficult to relate to any specific condition. These conditions are often poorly managed in current health services with frequent use of these services, but with poor clinical and cost effectiveness [1]. Further, several studies suggest that a small number of children with medical complexity account for a large proportion of health service costs [4, 5].

Great advances in medical and psychiatric care have inadvertently created individual disease or treatment “silos” [6, 7]. As information and knowledge accumulate, our health services have developed highly specialized and compartmentalized care, which is reinforced by specialty organizations, advocacy groups, disease management organizations, and government at all levels [7]. A single disease orientation is, however, often inefficient and causing care duplicity and inconsistency [6]. This organizing principle does not promote the coordinated multi-disciplinary efforts required to care properly for patients with multiple referrals and non-specific conditions.

In several countries worldwide, this fragmentation of care and services has been highlighted as a major challenge [1]. The disconnection of mental health services from the rest of the health services is highlighted as one of the present health care systems greatest shortcomings [8]. To manage complex patients with multiple referrals, non-specific conditions, medically unexplained symptoms, co- and multimorbid somatic and mental disorders, several multidisciplinary innovations have been, and are being, tried out. Such collaborative care models for adults with co-existing mental and somatic health complaints seem to be both clinical and cost-effective [9]. However, such coordinated services from pediatrics and mental health services are rare. Furthermore, research on how families with children with multiple referrals for mental and somatic conditions, perceive health services is scarce.

In an effort to improve services to better care for these patients, patient reported experience are paramount. This sort of feedback is intended to measure patient experiences with health services, focusing on areas such as user involvement, communication, availability and trust in health personnel. A systematic review of 55 studies indicated consistent positive associations between patient experiences, patient safety and clinical effectiveness across several different diseases, settings, measures and designs [10]. Patient experiences give important evidence for evaluating and developing health services through user involvement [11], and is in line with the idea of patient-centered health care, in Norway labelled “The Patient’s Health Services”. Patients reported experiences (PREM) of parent experiences of Norwegian pediatric [12], and child and adolescent mental health services (CAMHS) [13], respectively, have been published [14]. However, none of these addresses children with multi-morbidities or non-specific conditions receiving services provided simultaneously or in sequence by several hospital units.

It is our experience that many children with multiple hospital referrals, multimorbidities or non-specific conditions have a long and cumbersome road toward some sort of clarification of their condition, if they ever get one. The aim of the present study was to explore how to improve specialist health services for children with multiple referrals. Based on parent reported experiences of health services, we attempted to identify key areas of improvement.

## Method

### Design – setting

The present study was based on a selection of patients from our previous register study of complex care patterns [15]. The patient hospital data was included in the registry of Haukeland University Hospital, which is a regional hospital providing care across a wide range of clinical specialties, and covering a local catchment area of half a million inhabitants. In the area, only this hospital has specialist healthcare for children. Access to publicly funded specialist services is restricted in Norway with family doctors acting as “gate-keepers”. This study included patients aged 6–12 years who had at least one hospital episode from January 1, 2013 to December 31, 2015. We covered an inclusion period of 3 years because the ‘sample size’ of the study group of interest was unknown and a 3-year period would help ensure an adequate sample size. The minimum age for study inclusion was 6 years because all children in Norway undergo a medical check-up at their local child health clinic when they start school. An upper age limit of 12 years was set in this study. This upper age limit was decided due to ethical reason; adolescents in Norway are ethically defined as 13 years of age and other considerations must be done.

### Sample

**Fig. 1:**
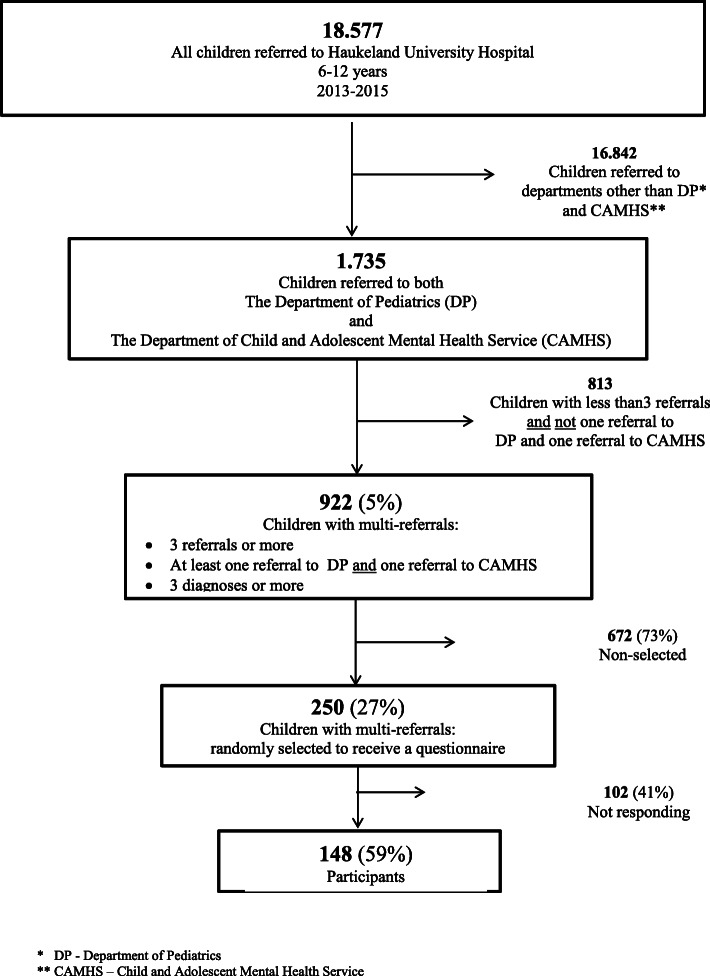
Population of parent reported experience with health services for children with multi-referrals

For the present study, we included patients with a higher probability of having complex care pathways as specified in the results of our previously published register study [15]. The group of interest (population) was specified as patients with the combination of three or more primary referrals in the 3 year period of inclusion (2013–2015) and with referrals to both somatic and mental healthcare (Fig. 1).

We refer to this criteria as multi-referrals. Next, at least one of these three referrals must be to child and adolescent mental health service and one to paediatric department. Of 922 patients identified, 250 (27%) were randomly selected for the present study (Fig. 1). Due to the explorative and descriptive purpose of the study with no a priori hypotheses, sample size calculation was not performed. Their parents/guardians were invited to answer questions regarding their experience with the health services they had received so far at Haukeland University Hospital.

### Measures

Parents/ guardians to children with multi-referrals and four professionals were invited to participate and develop the questionnaire. Five parents participated. All requested that the questionnaire was short and agreed on using a generic questionnaire, The Generic Short Patient Experiences Questionnaire (GS-PEQ), as the core of the questionnaire, but both parents and professionals requested two additional questions, respectively. Five of the original questions from GS-PEQ (item 1, 2, 11, 12 and 13) were modified due to comments from parents and health personnel, mainly to further specify the setting in question/adapt to the setting in question.

#### Parent reported experience (PREM)

The questionnaire was designed for the study and included a total of 14 questions (items). An adjusted version of a generic standardized questionnaire (GS-PEQ) was used [12, 13] and supplied with four items developed through user involvement from parents (2 items) and from health personnel at the hospital (2 items).

#### Standardized, generic questionnaire

The Generic Short Patient Experiences Questionnaire (GS-PEQ) (10 items) is a short, generic set of questions on user experiences with specialist health care and covers important topics for a range of patient groups [11]. GS-PEQ is the first generic, short questionnaire for collecting feedback on user experience across different types of services, patient and user groups. The development and psychometric properties of the GS-PEQ is described by Sjetne et al. [11]. We included eight out of the ten items in the Norwegian version of GS-PEQ, and reformulating five items, based on feedback from parent and health personnel. In addition we included two items (item 1 and 9) from specific adaptations of the GS-PEQ; Parent Experiences of Paediatric Care (PEPC) [12] and Parent experiences questionnaire for outpatient child and adolescent mental health services (PEQ-CAMHS Outpatients) [16]. Thus ten items (item 1, 2, 3, 5, 8, 9, 11, 12, 13 and 14) originate from the GS-PEQ and its discipline specific adaptations (PEPC and PEQ-CAMHS). The ten items from the GS-PEQ are recommended to be used alone or together with other instruments in quality assessment and have been developed and used to evaluate Norwegian health services [11]. The 14 items were scaled from “Not at all” to “To a very large extent” (1 to 5), in addition to the opportunity for answering “Not applicable” for any item the parents perceived not relevant (see Table 1).

**Table 1:** Parent reported experiences of health services for children with multi-referrals (*N* = 148): Descriptive information

*Item*^a^	Questions	N	*Mean*^b^	*SD*	Frequencies				
					1	2	3	4	5
i11	Did you experience the care at the hospital as useful for your child and family?	137	3.9	1.1	4.4	9.5	13.9	36.5	35.8
i13	Considering every consultation you have had with Haukeland University Hospital; did you experience being well cared for?	147	3.9	1.0	3.4	5.4	22.4	37.4	31.3
i1	Did you experience the wait time before assessment as acceptable?	142	3.5	1.1	5.6	9.2	33.1	33.1	19.0
i2	Did you experience the consultations at the hospital as accommodated to your child and family’s situation?	148	4.0	1.1	4.1	4.7	16.2	36.5	38.5
i3	Did the clinicians talk to you in a way that was easy to understand?	148	4.2	1.0	2.0	4.1	12.2	31.8	50.0
i4	Did you, overall, experience that the health personnel you met at the hospital were well informed about your child’s situation and medical history?	145	3.7	1.1	2.8	13.1	22.1	39.3	22.8
i5	Were you involved in any decisions regarding your treatment?	142	3.9	1.1	4.9	7.7	21.1	30.3	35.9
i6	Was the collaboration between health personnel and parents/child satisfactory?	148	4.0	1.0	0.7	11.5	15.5	35.1	37.2
i7	Did you as parents, experience being appreciated as important resources for your child by your therapist?	148	4.2	1.0	3.4	4.7	12.8	31.1	48.0
i8	Did you get sufficient information about your child’s diagnosis/afflictions?	143	3.7	1.2	4.2	11.2	26.6	24.5	33.6
i9	Were you told as much as you considered necessary about the purpose of the examinations?	146	4.0	1.0	2.1	5.5	15.1	41.8	35.6
i10	Did you receive adequate information about the following treatment of your child?	140	3.4	1.1	6.7	5.8	33.3	27.5	26.7
i12	Did you perceive the collaboration between the different departments at the hospital as well organized?	112	3.2	1.2	9.8	18.8	31.3	25.0	15.2
i14	Was the collaboration between the hospital and your general practitioner (GP) satisfactory?	100	3.3	1.3	12.0	17.0	24.0	27.0	20.0

^a^*Items* 1, 2, 3, 4, 5, 8, 9, 11, 12 and 13 originates from the Generic Short Patient Experiences Questionnaire or Parent Experiences of Paediatric Care (PEPC). Items 6 and 7 from parents, 4 and 10 from professions

^b^*Mean score of responses;* Response categories: value 1: Not at all; 2: To a little extent; 3: To some extent; 4: To a large extent; 5: To a very large extent

#### Specifically developed items

The standardized questionnaire was supplemented by questions proposed by both parents (2 items; 6 and 7 in Table 1), and health professionals (2 items; 4 and 10 in Table 1). This question generating process was intended to adapt the questionnaire to the setting in question, to ensure that the questionnaire addressed important aspects of parents’ experiences, and to ensure covering areas important to relevant health professionals.

#### Outcome and predictors

The outcome variables were the two variables: “Did you experience the care at the hospital as useful for your child and family?” (item 11) and “Considering every consultations you have had with Haukeland University Hospital; did you experience being well cared for?” (item 13) (Table 1), as these were thought to tap into more general aspects of the parents’ experience with the hospital. The remaining items were chosen as predictor variables.

### Data collection

The questionnaire was administered to parents/guardians of 250 randomly selected patients (see Fig. 1) aged six to 12 years, referred to Haukeland University Hospital. The questionnaire was written in Norwegian. If the families did not respond after 4 weeks a reminder was sent. If they did not respond to the reminder we called them after another 4 weeks to ensure that they had received the letters. Several parents asked if they could respond and answering the questions by phone, since this was more convenient for them.

### Statistical analysis

Descriptive analyses were used to describe the sample (mean, standard deviation, frequency) with SPSS version 24 [17]. The parents/guardians of 148 children responded. Analyses based on all actual variables for the study included *N* = 74 respondents. An important reason for “missing” information was that the subjects could respond with “Not applicable” at the questions. This response was mainly to the question regarding their perception of collaboration between the hospital and their general practitioner (GP) (variable 12: 36 missing; variable 14: 48 missing). Without these two questions, 118 subjects responded on all other questions, with 0–11 missing data in the other variables. The response “Not applicable” may mean different things for different respondents and regarding different questions. However, as we do not know this information this response was recoded to missing when analysing correlations and regression models. In order to use all available data, full information estimation (FIML) in Mplus version 8 was used [18], where predictors were included into the model within a latent variable framework [19]. This places distributional assumption on the predictors as well. The variables were explored and found to be relatively normally distributed (max skewness: 1.33). The maximum likelihood estimator with robust standard errors (MLR) handles non-normality in FIML. This assumes missingness to be randomly distributed (MAR) and not completely at random (MCAR), which is the case in ordinary analyses using the list wise deletion of cases with non-intact variables [20]. However, we cannot rule out the possibility of missingness being not at random (MNAR), as it is not possible to empirically test if missingness is MNAR or MAR. In order to explore content overlap between potential influential factors these were analysed with bivariate polychoric correlations. The final model regressed the two dependent variable on these predictors in logistic regression analyses. The Probit link function was used in order to base the categories on an underlying latent variable [19]. Both dependent variables were included in one model, with simultaneously testing of all predictors.

### Ethical considerations

The study has been assessed by the Regional Committee for Medical Research Ethics by means of presentation assessment (Ref. nb: 2017/886). According to the committee, additional approval was not required for this quality assurance and evaluations project as a part of health services activities, as the project does not include changes in practice and the data is anonymous. The respondents were informed that participation was voluntary and that the data was anonymous.

## Results

### Participants

Of the 250 parents invited, 148 (59%) participated; 32 responded by mail and 116 by telephone. Out of the 102 who did not reply, seven replied that their child was in foster families/care and thus did not feel qualified to participate. Six invitations were returned due to incorrect address. Mean age of the 148 children was 82 months (SD = 31.5), almost 7 years, and 93 (63%) boys. We did not have permission to compare responders and non-responders.

### Parent reported experience with health services

The 14 questions/items are presented in Table 1 with mean, standard deviation and response frequencies. The single item response mean scores ranged from 3.18 (3 = “To some extent”) to 4.42 (4 = “To a large extent). The majority of items had mean scores in the range 3 to 4 on the scale from 1 to 5 (best possible score/“To a very large extent”). Almost one in three parents (*N* = 48 (32%)) responded “not applicable” to the question regarding their perception of collaboration between the hospital and their general practitioner (GP). The frequencies showed most variables to be somewhat skewed with most responses in the highest categories, while other variables showed most responses in the lowest categories.

In Table 2 bivariate correlations between the variables, defined as the 14 items in the questionnaire, are presented. The correlations between the different predictors were found to be moderate to high, however, with a few low in magnitude.

**Table 2:** Model estimated means, standard deviations (SD) for all variables, and bivariate polychoric correlations between predictor variables (under diagonal) (*N* = 148)

*Item*	*Mean*	*SD*	Correlations											
			i1	i2	i3	i4	i5	i6	i7	i8	i9	i10	i12	i14
i11	3.91	0.75												
i13	3.88	1.19												
i1	3.51	1.07	1											
i2	4.01	1.05	.38	1										
i3	4.24	0.95	.21	.66	1									
i4	3.66	1.05	.39	.64	.53	1								
i5	3.85	1.14	.36	.61	.57	.56	1							
i6	3.97	1.02	.49	.77	.68	.67	.73	1						
i7	4.16	1.04	.40	.60	.65	.63	.73	.79	1					
i8	3.73	1.16	.45	.61	.56	.57	.50	.70	.58	1				
i9	4.04	0.95	.28	.61	.62	.58	.65	.70	.61	.63	1			
i10	3.39	1.12	.43	.52	.45	.42	.49	.54	.46	.64	.48	1		
i12	3.18	1.19	.54	.58	.40	.60	.45	.53	.43	.55	.56	.51	1	
i14	3.27	1.29	.22	.41	.19	.48	.35	.52	.41	.44	.51	.48	.48	1

*Item* 1, 2, 3, 4, 5, 8, 9, 11, 12 and 13 are questions originating from the Generic Short Patient Experiences Questionnaire or Parent Experiences of Paediatric Care (PEPC). Items 6 and 7 from parents and10 and 14 from professions

*Mean* and *SD* (standard deviation) values are model estimated under FIML

Table 3 shows bivariate correlations and regression results between the item 11 and 13 as outcome variables and predictors which are the 12 other items. The analyses showed that higher levels in all predictors were related to higher levels in the two outcome variables, with some variations in the associations (i11: .30–.71; i13: .56–.73). Using all predictor variables in the multiple regression resulted in prediction models with high levels of explained variance. The outcome “Did you experience the care at the hospital as useful for your child and family?” (i11) was predicted with 65%, and the variable “Considering every consultation you have had with Haukeland University Hospital; did you experience being well cared for?” (i13) with 71%. The first variable (i11) was statistically significantly predicted by the following variables: i2 (consultations accommodated to your child and family’s situation), and i10 (receive adequate information about the following treatment of your child), with the strongest relation being the i2 variable. The variable “experience being well cared for” (i13) was statistically significant predicted by i1 (experience the wait time before assessment as acceptable), i2 (the treatment received as suited to your child and family’s situation), and i12 (Did you perceive the collaboration between the different departments at the hospital as well organized?). The i2 relationship was also here the strongest one. However, other variables not being statistically significant may be partly related to the two outcome variables with their overlap with these predictors (correlations, Table 2), and the high level explained variance of the total models.

**Table 3:** Bivariate polychoric correlations between outcome (i11 and i13) and predictor variables, and multiple logistic regression standardized results between outcome and predictor variables (*N* = 148)

	Bivariate correlations						Multiple Logistic regression models					
	i11			i13			i11			i13		
	*r*	*CI*	*P*	*r*	*CI*	*P*	*β*	*CI*	*P*	*β*	*CI*	*P*
i1	.30	.14–.47	<.001	.56	.44–.69	<.001	−0.15	−0.33-0.02	.072	0.16	0.05–0.28	.007
i2	.71	.62–.80	<.001	.73	.65–.81	<.001	0.25	0.06–0.45	.013	0.27	0.09–0.44	.004
i3	.56	.44–.69	<.001	.59	.48–.69	<.001	−0.03	−0.19-0.13	.724	0.10	−0.06-0.25	.232
i4	.59	.46–.72	<.001	.61	.51–.72	<.001	0.07	−0.13-0.27	.469	−0.02	− 0.19-0.15	.855
i5	.65	.53–.77	<.001	.59	.47–70	<.001	0.14	−0.01-0.30	.075	0.03	−0.21-0.27	.806
i6	.69	.59–.79	<.001	.69	.60–.78	<.001	0.03	−0.21-0.27	.799	−0.10	−0.32-0.13	.417
i7	.65	.54–.77	<.001	.63	.51–.75	<.001	0.14	−0.02-0.30	.072	0.10	−0.10-0.30	.311
i8	.63	.52–.75	<.001	.67	.57–.77	<.001	0.09	−0.10-0.28	.334	0.10	−0.05-0.25	.188
i9	.62	.50–.74	<.001	.67	.57–.76	<.001	0.04	−0.19-0.26	.735	0.11	−0.08-0.29	.264
i10	.66	.55–.77	<.001	.61	.49–.73	<.001	0.26	0.09–0.43	.004	0.09	−0.06-0.23	.236
i12	.56	.42–.70	<.001	.68	.57–.78	<.001	0.10	−0.11-0.32	.343	0.20	−0.01-0.40	.047
i14	.48	.31–.65	<.001	.56	.43–.69	<.001	0.02	−0.18-0.22	.856	0.16	−0.03-0.34	.119
R^2^							.63			.70		

*r: correlations, β: standardized regression weights, CI: 95% confidence intervals for the estimates*

*Item* 1, 2, 3, 4, 5, 8, 9, 11, 12 and 13 are questions originating from the Generic Short Patient Experiences Questionnaire or Parent Experiences of Paediatric Care (PEPC). Items 6 and 7 from parents and 10 and 14 from professions

## Discussion

This is, to our knowledge, the first study to assess parent experiences with concurrent pediatric and psychiatric health services for children with multiple hospital referrals. In this study, parents report relatively positive experiences with health services, which is in line with previous studies [13, 16]. Some, however, claim that parent reports of satisfaction is, at best, an overestimation of actual satisfaction [21]. Thus, looking at specific care elements to improve services, instead of looking for levels of experience, might be more feasible. The results show high levels of predictor relations and high levels of explained variance in both multiple regression models. Based on a conceptual understanding of the variables, this indicate that predictors other than those found to be statistically significant also are relevant for the parents overall experience. Both statistical significant, but also other related predictors, are seen as different aspects of service quality and consumer satisfaction. The most important findings are represented by four relationsships. The perception of a reasonable wait time before treatment (i1), accommodating consultations at the hospital to the child and family’s situation (both in terms of practicalities and in terms of the child’s condition (i2), providing adequate information about the following treatment (i10), and collaboration between different departments at the hospital (i12), as they seem to be associated with parents overall experience.

### Wait time

Several studies have linked perception of wait times to dissatisfaction [22]. Previous studies also suggest unwanted waiting is related to less positive parental experiences with health services [12, 23]. One could speculate if the perception of the wait before treatment/consultation increase parental expectations for the consultation, thus also increasing the odds for disappointment. Maybe a long wait might be compensated by a very useful consultation or a successful treatment? Some studies suggest that positive experiences with consultations can mitigate negative responses to perceived wait time [24].

### User involvement/accommodating consultations

Accommodating consultations at the hospital to the child and family’s situation seem to be related to the overall experience, thus highlighting the importance of tailoring treatment and services to patients and families. This might be difficult in a compartmentalized and highly specialized health services, as this mode of organization impede a more holistic understanding and treatment of the child, not only caring for a single specific condition, and attempting to compartmentalize and single out several distinct, definable, and independently treatable conditions. Compartmentalization and super-specialization might result in parallel and fragmented care, and not treating the patient as a whole. Others also suggest [25] tailored health services interventions for medically complex children and their families.

### Communication/information about treatment

Similar studies [16] in CAMHS in Norway also support focusing on improving communication and user involvement to improve parent experiences of health services. Good and effective communication has been highlighted as a key element of integrated physical and mental health care [1, 26]. This includes the way information is provided by health personnel and received by families, giving patient and families comprehensible and personalized information about their condition and treatment process. According to our study, receiving adequate information about the child’s following treatment seemed to be positively associated with overall experience of health services for children with multi-referrals. The item concerning receiving information about the subsequent treatment was the fifth lowest rated item of parent experiences in our study. The frequency distribution indicates that some parents either did not receive adequate information, or did not understand the information, about subsequent treatment. This could also be an indication of a lack of clarification of the child’s condition(s) and corresponding recommended treatment. In many cases getting information about treatment hinges on some sort of clarification of the child’s condition, as treatment in many cases ensues specific diagnoses. There is at prevalent trend in western health services to develop evidence-based diagnostic and treatment protocols for specific diagnoses [6], but this procedure falls short of devising adequate or useful treatment for children with less definable, non-specific conditions. Some clusters of complaints might be trans-diagnostic, spanning several diagnostic categories, making it harder to divide in manageable parts or diagnoses. With this in mind, considering a more “patient-centered” [6] or “function-oriented” approach versus the more prevalent “disease-oriented” approach might provide more suitable and flexible treatment options for children with multi-referrals to specialist health services.

### Collaboration between different departments at the hospital

This finding highlights multi-referrals need for coordinated specialist support [1], and tentatively supports considering abandoning a single disease orientation for a more holistic and interdisciplinary approach.

### Need for interdisciplinary collaboration/coordination of services

To address the areas of improvement resurfacing in this study, we propose interventions meant to help interdisciplinary and complementary assessment and clarification of conditions in children with multi-referrals to both mental and somatic specialist services, and especially those with non-specific conditions. This to make communication and delivering adequate information about conditions and following treatment easier, collaboration between hospital departments easier and to more readily accommodate the treatment to the patient’s unique situation or conditions, irrespective of the condition being specific or non-specific, and irrespective of which department at the hospital the child currently is in. We believe that when it comes to children with multi-referrals and non-specific conditions, accommodating treatment and giving useful information about the treatment is especially challenging for health personnel. These tasks are challenged by the non-specific nature of the patient’s complaints and the compartmentalization of modern day health services, and are thus in special need of attention and improvement. In this regard, the co-occurrence of mental and physical diseases in childhood has been highlighted as a major public health challenge in need of well-coordinated and integrated interdisciplinary approaches [2]. Sasseville, Chouinard and Fortin [27] point to holism as a philosophical underpinning for such multimorbidity interventions, while others propose adhering to a more biopsychosocial model of understanding versus a biomedical one [28]. According to the World Health Organization [29] one of the five common shortcomings of today’s health care delivery is fragmented care; where super-specialization of health services and the narrow focus of many disease control programs discourage a holistic approach to individuals and families, and disregards the need for holism and continuity in care. It is our hypothesis that interdisciplinary and complementary teams with a holistic underpinning, will make accommodation of treatment and communication regarding this treatment more meaningful for parents of, and children with multi-referrals to specialist health services. A common assessment process is previously highlighted as a starting point for care coordination [1]. Including different hospital departmentsin an interdisciplinary and complementary team gathering around the child, might increase families´ perception of continuity of care [30]. This might also make collaboration across different disciplines and service levels easier, more productive and meaningful. It also has the potential to reduce wait time by giving an interdisciplinary assessment of the child’s condition, potentially reducing the risk for care and assessment duplicity. Previous studies suggest that families with more complex conditions are more vulnerable for experiencing discontinuity in services [31]. Insufficient tailoring of health services represent a major shortcoming in our health services, and contributes to poor outcomes for patients and for health service investments. Thus, developing tailored health services for patients with compound conditions does not only have the potential to improve patient and family lives, but also public spending.

### Methodological issues

Response rates in user satisfaction surveys in CAMHS are generally low, ranging from a median of 33% on mailed surveys to 55% on telephone interviews [21]. Thus, a response rate in the present study of 59% appears acceptable. As most of the respondents in this survey replied per telephone (73%), this could have increased the response rate, lead to more positive feedback [32] and possibly to an overestimation of mean experiences. Analysis gave, however, only one statistically significant difference in responses, where respondents by telephone had statistically significantly higher ratings than mail respondents, on item 3, ie. if the clinicians talk to you in a way that was easy to understand. This might reflect an actual difference in experiences, or possibly be a result of the social desirability bias [33]. The respondents might report more positive experiences, because they feel this will be more acceptable to the services in question, especially if representatives from the services in question themselves are collecting feedback from users [33]. The analyzed sample size is related the risk of type 2 errors, indicated by relatively wide confidence intervals (i.e. i5; β = 0.14, CI = -.01.30). This implies that several results would reach statistical significance in samples of higher size, given all other information being equal. Adapting existing validated questionnaires might reduce the internal validity of the results. We involved parents and professionals to ensure adequate specificity and covering themes relevant to them. The diversity of conditions treated in mental health services makes it difficult to develop universal instruments for measuring patient and parents/guardians satisfaction [34]. Hence, some sort of adaptation of generic instruments may be necessary to develop instruments specific and sensitive enough to capture existing differences in experiences. Few studies consider children’s own perception of quality of care, and preliminary studies suggest that children and adolescents are less satisfied with mental health services than their parents, showing a weak to moderate correlation between child and parent satisfaction [34]. However, for the children in our sample, the decision to seek referral mainly lies with the parent. Studies also suggest that parent satisfaction is more strongly related to functional improvement and reduction of symptoms, than the satisfaction of the child [34]. The questionnaire was only available in Norwegian, possibly excluding people less fluent in Norwegian. The parents had to volunteer to participate, possibly excluding very dissatisfied parents [21]. All of our postal non-responders, that we were able to reach, were offered to participate via telephone interview. The number of respondents are also relatively low, and there might be important variations in the children’s conditions. The fact that we included the response “not applicable” in the questionnaire, sadly forced us to treat this as missing data in the analyses, given its ambiguity. The use of this response option is particularly prevalent in the responses to items 12 and 14 (see Table 1) regarding perception of the organization of the work at the hospital (i12) and the perception of collaboration between the hospital and their general practitioner (GP) (i14). Out of 148 responders; 9 parents responded “not applicable” to item 12 and 27 did not respond (missing), while 47 parents responded “not applicable” to item 14 and 1 did not respond (missing). The high level of “not applicable”-responses is in line with previous studies using GS-PEQ [11]. Without items 12 and 14, 118 subjects responded to all other questions. Parent reported experience measures of health services have several methodological challenges; low response rate, high level of satisfaction and lack of validated instruments/surveys [21]. We have used validated instruments, we have a relatively reasonable response rate, but the results in general reflect high levels of satisfaction/positive experiences of services. As a result, we have emphasized, areas of improvement rather than levels of experience.

## Conclusion

The study support tailored interdisciplinary innovations targeting reducing wait time, accommodating consultations, giving adequate information about treatment and strengthening collaboration between different departments within specialist health services for children with multi-referrals to somatic and mental specialist health care services presenting with non-specific conditions. Future studies addressing children’s experience with specialist health services are recommended.

## Data Availability

Restrictions apply to the availability of these data, which were used under license for the current study, and so are not publicly available. Data are however available from the authors upon reasonable request and provided we obtain additional permission from the participants to make the data available to others.

## Abbreviations

PREM: Patient Reported Experience Measures
CAMHS: Department of Child and Adolescent Mental Health Services
DP: Department of Paediatrics
GS-PEQ: The Generic Short Patient Experiences Questionnaire
PEPC: Parent Experiences of Paediatric Care
PEQ-CAMHS: Parent experiences questionnaire for outpatient child and adolescent mental health services
SPSS: Statistical Package for the Social Sciences
GP: General Practitioner
PU: Department of Pediatrics
FIML: Full information estimation
MLR: Maximum likelihood estimator with robust standard errors
MAR: Missingness randomly distributed
MCAR: Missingness not completely at random
MNAR: Missingness not being at random
Ref: Reference
nb: number
SD: Standard Deviation
N: Population Size
i: Item
r: Correlations
β: standardized regression weights
CI: 95% confidence intervals for the estimates
